# Current status of nontuberculous mycobacterial surgery in Japan: analysis of data from the annual survey by the Japanese Association for Thoracic Surgery

**DOI:** 10.1007/s11748-015-0594-z

**Published:** 2015-10-03

**Authors:** Yuji Shiraishi

**Affiliations:** Section of Chest Surgery, Fukujuji Hospital, 3-1-24 Matsuyama, Kiyose, Tokyo 204-8522 Japan

**Keywords:** Nontuberculous mycobacteriosis, Tuberculosis, Tuberculoma, Resectional surgery

## Abstract

**Objective:**

The prevalence of pulmonary disease caused by nontuberculous mycobacteria (NTM) has been increasing in Japan. Adjuvant resectional surgery is often recommended to lessen disease progression when the response to drug therapy is poor. In all likelihood, as affected cases of NTM disease increase, so will the number of operations. The goal of this study was to determine the current status of NTM surgery in Japan by analyzing data from the annual survey of the Japanese Association for Thoracic Surgery (JATS).

**Methods:**

Data were obtained from annual surveys conducted between 2008 and 2012. The annual number of operations for pulmonary NTM disease was tabulated nationwide and in each region (Hokkaido, Tohoku, Kanto, Tokyo, Chubu, Kinki, Chugoku/Shikoku, and Kyushu). For comparison, the numbers for pulmonary tuberculosis and tuberculoma operations were also obtained.

**Results:**

The annual number of operations for pulmonary NTM disease nationwide increased each year between 2008 and 2012: 292 (2008), 323 (2009), 452 (2010), 440 (2011), and 514 (2012); an overall increase of 76 %. Conversely, the annual numbers of operations for pulmonary tuberculosis were stable: 145 (2008), 181 (2009), 117 (2010), 113 (2011), and 107 (2012), as were the annual numbers of operations for tuberculoma: 386 (2008), 341 (2009), 320 (2010), 390 (2011), and 351 (2012).

**Conclusion:**

Data from the JATS annual survey demonstrate a steady increase in the number of NTM surgeries in Japan. General thoracic surgeons will continue to increasingly encounter NTM patients who are candidates for surgery until a magic bullet against NTM disease is available.

## Introduction

Since the advent of potent anti-tuberculous drugs, the prevalence of pulmonary tuberculosis has been decreasing in Japan. In contrast, it is thought that the prevalence of pulmonary disease caused by nontuberculous mycobacteria (NTM) has been increasing. The estimated incidence rate of pulmonary NTM disease in Japan has increased from 1.5 per 100,000 in 1985 to 3.52 in 1997 [1]. NTM disease is usually indolent, but can result in extensive parenchymal destruction, causing respiratory failure and vulnerability to airway infection, which can be fatal [2]. According to the statement published by the American Thoracic Society (ATS)/Infectious Diseases Society of America (IDSA) in 2007, the primary treatment for pulmonary NTM disease is chemotherapy using a multidrug regimen [3]. However, chemotherapy has limited efficacy in pulmonary diseases caused by the majority of NTM species. Therefore, the statement proposed a multidisciplinary treatment approach, i.e., combined multidrug treatment regimen and adjuvant resectional surgery, for patients with intractable pulmonary NTM disease. The Japanese Society for Tuberculosis (JST) published guidelines for surgical intervention of pulmonary NTM disease in 2008, which recommended a multidisciplinary treatment approach consistent with the 2007 ATS/IDSA statement [4]. The higher the prevalence of pulmonary NTM disease, the more likely the number of operations for this disease will increase. This study was designed to clarify the current status of NTM surgery in Japan by analyzing data from the annual survey of the Japanese Association for Thoracic Surgery (JATS).

## Materials and methods

Data from the JATS annual survey conducted between 2008 and 2012 were used in this analysis. The year 2008 was used as a starting point because it was the year that survey questionnaires added the category of pulmonary NTM disease. The number of survey questionnaire forms that were sent out to officially certified institutions nationwide and their response rates in each year were as follows: 785/95.3 % in 2008 [5], 798/96.5 % in 2009 [6], 787/97.2 % in 2010 [7], 790/95.6 % in 2011 [8], and 802/96.9 % in 2012 [9]. The total numbers of general thoracic operations in each year were 61,315 in 2008 [5], 65,897 in 2009 [6], 67,960 in 2010 [7], 69,223 in 2011 [8], and 72,899 in 2012 [9]. The annual number of operations over the 5-year period for pulmonary NTM disease, regionally (Hokkaido, Tohoku, Kanto, Tokyo, Chubu, Kinki, Chugoku/Shikoku, and Kyushu) and nationwide, was extracted from the JATS annual survey database that comprises anonymized raw data. Data on the total number of operations for pulmonary tuberculosis and tuberculoma were also obtained for comparison.

## Results

Nationally, the number of operations for pulmonary NTM disease in 2012 increased 76 % over the number performed in 2008 (Table 1). The ratio of the number of operations for pulmonary NTM disease to the total number of general thoracic operations also increased from 0.5 % (292/61,315) in 2008 to 0.7 % (514/72,899) in 2012. Regionally, an increase in NTM surgeries was particularly noted in Tohoku, Kanto, Tokyo, Chubu, Kinki, and Chugoku/Shikoku. In contrast, the annual number of operations performed for pulmonary tuberculosis (Table 2) and tuberculoma (Table 3) was stable during the 5-year period.

**Table 1:** Annual trend of number of national and regional operations for pulmonary nontuberculous mycobacterial disease

Region	2008 [5]	2009 [6]	2010 [7]	2011 [8]	2012 [9]
Hokkaido	12	18	26	16	13
Tohoku	16	19	37	28	39
Kanto	54	62	96	72	86
Tokyo	33	57	53	72	90
Chubu	40	58	69	83	78
Kinki	72	45	68	67	95
Chugoku/Shikoku	32	46	60	52	75
Kyushu	33	18	43	50	38
Nation (total)	292	323	452	440	514

**Table 2:** Annual trend of number of national and regional operations for pulmonary tuberculosis

Region	2008 [5]	2009 [6]	2010 [7]	2011 [8]	2012 [9]
Hokkaido	5	13	6	8	2
Tohoku	4	4	4	6	1
Kanto	20	33	29	20	23
Tokyo	20	42	13	23	19
Chubu	8	19	17	9	14
Kinki	49	44	19	27	23
Chugoku/Shikoku	18	12	11	10	8
Kyushu	21	14	18	10	17
Nation (total)	145	181	117	113	107

**Table 3:** Annual trend of number of national and regional operations for tuberculoma

Region	2008 [5]	2009 [6]	2010 [7]	2011 [8]	2012 [9]
Hokkaido	24	34	17	18	14
Tohoku	30	30	38	28	19
Kanto	73	77	64	81	96
Tokyo	57	45	35	55	30
Chubu	53	48	31	46	49
Kinki	89	67	87	77	86
Chugoku/Shikoku	27	26	24	47	34
Kyushu	33	14	24	38	23
Nation (total)	386	341	320	390	351

## Discussion

Nontuberculous mycobacteria are environmental organisms found in soil and water that can produce opportunistic lung infections. The true incidence and prevalence of pulmonary NTM disease remains unclear because this disease is not communicable and has not been designated a reportable disease by the Ministry of Health, Labour, and Welfare in Japan. However, reports suggest that the prevalence of pulmonary NTM disease has been increasing [1, 2]. Of 120+ species of mycobacteria classified as NTM, only a few are known to lead to pulmonary disease [3]. In Japan, members of the *Mycobacterium avium* complex (MAC) are the most common source of pulmonary NTM disease [10]. *Mycobacterium kansasii* is the second most common NTM, but the incidence of *M. kansasii* pulmonary disease is much lower than that of pulmonary MAC disease [10]. Furthermore, a *M. kansasii* pulmonary infection is the only NTM disease that can be successfully treated with a multidrug regimen alone [3, 10]. Therefore, in daily clinical practice, physicians are more likely to be consulted about surgical intervention for pulmonary MAC disease.

The problem is that there is no specific agent for the treatment of pulmonary MAC disease. Although the ATS has recommended a multidrug regimen employing the newer macrolides (clarithromycin or azithromycin), rifampicin or rifabutin, ethambutol and, if necessary, injectable aminoglycoside (streptomycin) [11], the efficacy of this program remains limited. Kobashi and Matsushima reported that the combined therapy for pulmonary MAC disease recommended in the ATS [11] and the JST [12] guidelines produced unsatisfactory results compared to the clinical effect of the standard multidrug regimen for the treatment of pulmonary tuberculosis [13]. The 2007 ATS/IDSA statement recommended a multidisciplinary treatment approach to prevent disease progression: a combination of chemotherapy and adjuvant resectional surgery for patients with intractable NTM disease [3]. The JST guidelines also recommended this multidisciplinary treatment approach [4].

In this study, data from the JATS annual survey revealed a steady increase in the annual number of operations performed in Japan for pulmonary NTM disease. The overall number increased by 76 % in 5 years. However, the annual number of operations for pulmonary tuberculosis was stable, as was the annual number of operations for tuberculoma in the country. The total number of general thoracic operations increased during the study period. However, the increase in the number of operations for pulmonary NTM disease cannot be explained simply by the increase in the number of total operations, because the ratio of the number of operations for pulmonary NTM disease to the total number of general thoracic operations also increased. The recent increase in the number of operations for pulmonary NTM disease may reflect the fact that the prevalence of pulmonary NTM disease has been increasing in Japan and that the 2007 ATS/IDSA statement and the 2008 JST guidelines have encouraged Japanese physicians to liberally apply adjuvant resectional surgery to patients with pulmonary NTM disease. This trend is expected to continue indefinitely. Japanese General Thoracic surgeons will continue to increasingly encounter NTM patients who are candidates for resectional surgery until a magic bullet against this intractable disease is available. A notable increase in the number of operations for pulmonary NTM disease was seen in six of the eight regions. Further investigation is needed to clarify a regional distribution of patients with pulmonary NTM disease in Japan.

Data from the JATS survey did not contain crucial information (e.g., as patient characteristics, including age, gender, NTM species, and disease type) that would have allowed a more precise analysis of the current status of NTM surgery. The data also lacked information on the duration of pre-operative chemotherapy, the type of pulmonary resection, and the duration of post-operative chemotherapy. Pulmonary MAC disease predominantly occurs in middle-aged women, and consists of two radiographically characteristic features: fibrocavitary disease and nodular/bronchiectatic disease [3]. Although MAC disease is the NTM disease most likely to require pulmonary resection, other less common NTM diseases, such as *Mycobacterium abscessus* disease, which is notoriously resistant to chemotherapeutic regimens, may also be subject to resectional surgery [14]. According to the JST guidelines, surgery should be performed after 3–6 months of chemotherapy in expectation of the limited but possible efficacy of chemotherapy and the reduction of bacterial load [4]. The procedure of choice is pulmonary resection; segmental or greater resection is warranted if either disseminated foci surround the affected lesions or if the lesions disseminate into airways because the lesions can extend through the airways. Chemotherapy should be continued for at least 1 year after surgery in accordance with the ATS/IDSA guidelines for medical therapy (i.e., for at least 1 year after sputum conversion) [3], and on the basis of previous experience. The National Clinical Database established in 2011 can provide information about a patient’s age, gender, and type of pulmonary resection, but other relevant information remains missing. To perform a more in-depth analysis of the current status of NTM surgery, we need to conduct a national survey on this disease.

## Conclusions

The JATS annual survey revealed a steady increase in the annual number of operations for pulmonary NTM disease in Japan. Japanese General Thoracic surgeons will continue to increasingly encounter NTM patients who are candidates for resectional surgery until a magic bullet against this intractable disease is available. A national survey focusing on this disease is needed for more in-depth analysis of the current status of NTM surgery.
